# From Our Correspondents

**Published:** 1983

**Authors:** 


### From Our Correspondents

### Chair of Orthopaedic Surgery

Mr. David Fuller has been appointed as the first Professor of Orthopaedic Surgery at Bristol University and will start in September 1983. This simple statement hides a very remarkable achievement. When Michael McCormack and his colleagues resolved to establish a Chair in their speciality some years ago, they realised that the venture was beyond the financial scope of the University and set up a special Appeal. Sir Reginald Verdon-Smith (Pro-Chancellor) became President of the Appeal, Earl Waldegrave KG and eleven others agreed to serve as Vice-Presidents and Mr. John Pool was appointed Chairman of Trustees. The greatest coup of all was to secure the support of HRH The Prince of Wales as Patron of the Appeal. The Prince stressed that Bristol was the only University with a Medical Faculty in the Duchy of Cornwall, and the importance of the regional contribution was underlined by the sonorous title: The Bristol and West of England Appeal for a Chair in Orthopaedic Surgery at the University of Bristol.

After accepting the academic case for an Orthopaedic Professor, the University set the daunting target of half a million pounds to fund the Chair in reasonable perpetuity. The Prince of Wales officially launched the Appeal during a visit to the Bristol Royal Infirmary on 6th November 1980. Two years later, thanks to the enterprise and effort of those involved, the target had drawn close enough for the University to advertise and appoint the Foundation Professor. In recognition of the major benefaction he will be known as the Zimmer Deloro Professor of Orthopaedic Surgery.

Now a Consultant Surgeon at Oxford and an international authority on clubfoot, Mr. Fuller was once an Orthopaedic Registrar in Bristol. Bristol and Weston District Health Authority have generously agreed to provide the crucial support of a Consultant Senior Lecturer from 1984. The two consultants will become members of the University Department of Surgery, together with the existing Lecturer in Orthopaedic Surgery.

This is a fine success story, especially coming at a time when the University of Bristol is facing serious financial difficulties. A public appeal has provided the funds for the first established Chair in a surgical speciality. How Hey Groves would have applauded! An Honorary Professor himself and first Editor of the *British Journal of Surgery*, he was the founder of orthopaedics in Bristol. Most fittingly, the Prince of Wales returned to Bristol on 19th November 1982 to a Gala Performance at the Theatre Royal, there to meet the man whose Chair he helped to create.

R.C.N.W.

### General Practice

General practice in Bristol has come of age – at last Bristol medical professors have recognised the need for a University Department of General Practice. Bristol holds the questionable distinction of being the last provincial medical school in the United Kingdom to have no department of general practice.

What will such a department do? Its prime task will be to organise the undergraduate teaching in the special characteristics of general practice care: the different spectrum of illness that exists outside teaching hospitals, the benefits and problems of continuity of care, the special features illness takes on in the context of the family and above all the satisfaction and challenge experienced by doctors who commit themselves to care for an individual and his family in sickness and in health.

The department will also have a major role in research. General practice research is in its infancy and university departments will have a major role in raising standards of such research, co-operating with other disciplines and challenging many long held beliefs that have been generated within teaching hospitals that may have little relevance to life in the community.

There remains one major problem – financing such a department. It will be no easy task.

M.W.

### Royal College of Obstetricians and Gynaecologists

The *Royal College of Obstetricians and Gynaecologists* has been busy. Hard on the heels of the Report on Training for Obstetrics and Gynaecology for General Practitioners by a Joint Working Party of the R.C.O.G. and R.C.G.P. has come the Report of the R.C.O.G. Working Party on Antenatal and Intrapartum Care, a discussion document on Manpower in Obstetrics and Gynaecology, and the report of the committee set up to discuss subspecialisation in Obstetrics and Gynaecology.

Although the discussions of each committee took place in isolation from those of the others, these reports are, in the main, complementary to one another. Among the recommendations which can be distilled from their reports are:

1. That the 'functional integration of all maternity services within districts should be vigorously pursued' and that the necessary facilities, vocational training opportunities and continuing education be provided for all involved.
2. Integration of maternity care should be the responsibility of a Maternity Services Liaison Committee.

3. Medical and midwifery staff need to provide 'a flexible attitude to antenatal and intrapartum care, and a willingness to consider alternative approaches as ideas change'.
4. The scope of the problems faced in the specialty and the newer methods available for their detection and treatment mean that no one consultant obstetrician and gynaecologist is totally competent to manage everything at all times.

There is, therefore, a need for a small proportion of the consultants to become subspecialists in such areas as gynaecological oncology, 'reproductive medicine' (e.g. infertility and endocrinology) and perinatal medicine. A further proportion, but still the minority, will take up special interests in these areas but at a less intense level.

It is, however, around the manpower proposals that the current debate centres. Faced with an excess of senior registrars looking for consultant jobs, possibly panicked by the Short Committee Report, among the problems the College is trying to tackle are: should we have a consultant-led or a consultant-based service; the increasing proportion of women doctors; the inadequate training offered overseas graduates who form 75% of the registrar establishment, and the unequal distribution of workload throughout the regions. Whatever the merits (or otherwise) of their proposals they have, like the Short Committee, failed to consider the financial consequences of their recommendations which will be enormous. In our present economic plight our paymasters have the 'Nelson's eye' approach; that is, they see the recommendations which will save money and turn a blind eye to developments which have 'revenue consequences'.

Whatever reservations one has about any of their recommendations the R.C.O.G. must be congratulated in at least addressing themselves to our most pressing problems in a constructive manner. Let us hope that once the ostrich has removed its head from the sand, its neck is not so long as to make it disappear in the clouds.

G.S.

**Ham Green Hospital**

*Community*

For many years the people of Pill and the surrounding area have, rightly, looked upon Ham Green as their hospital. The recent re-organisation of the Health Service has resulted in an every closer link between hospital and the community, following the appointment of our new Nursing Administrator (Mr. R. Cooper) who is responsible for both Hospital and Community Nursing Services.

*Coronary Care Unit*

With generous financial help from both the District Management Team and many voluntary organisations and individuals we have been able to upgrade all our old Coronary Care equipment and will be showing our appreciation by holding an Open Day to which all who have contributed will be invited to see the equipment they have helped to supply.

*Education*

Postgraduate activities for both Hospital Doctors and General Practitioners continue to increase and I, as Clinical Tutor, am very grateful for all the support I get from my colleagues. We have started to have undergraduate students attached to some of the Consultants, and all our doctors, both Senior and Junior, have appreciated their enthusiasm and the challenge of teaching, and hope the practice will continue, and hopefully, even extend. Grateful thanks must be extended to the University, our own (Southmead) Clinical Dean (Dr. Francis Page) and the Consultants for this opportunity to take part in undergraduate teaching.

*Building*

Several major building projects are underway in the hospital. These include a new kitchen and dining room and new boiler. There are healthy signs in the continued development of the hospital.

D.W.W.

**Cossham Medical Society**

The Cossham Medical Society which meets each month at Frenchay Hospital was recently entertained to a fasinating talk on Dr. Jenner by Canon Gethin-Jones, of Berkeley. That the world was relieved of the scourge of Smallpox by this great doctor was the message of this enthusiastic raconteur. The world also seems more aware of the debt owed to him, for it is extraordinary how many statues, street names and even shops have been named after Jenner, apart from the gifts bestowed on him during his life time. An appeal is at present being launched to help refurbish the Jenner Memorial Museum at the Chantry, Berkeley. It is salutary to us who live in the locality that a Japanese business man has donated half a million pounds to the appeal. Is there anyone closer to home who would care to match such a gift? If so please contact Dr. C. Burns-Cox at Frenchay Hospital!

G.T.

**Southmead Hospital**

'The main buildings are approached by an avenue lined by mature pink flowering horse chestnuts. At the top there is a beautiful *Cedrus deodara* with a large spreading Paulownia (foxglove tree) and the rare November blossoming purple flowers of the Callicarpa bush. By the chapel an elegant Gingko (maiden hair tree) contrasts with the deep green leaves of a *Viburnum rhyditophyllum* and the even deeper colours of a large Irish Yew. Observe how the lovely Wisteria sets off the grey stone of the main buildings, the bank of tree paeonias in front of the Hall and the newly planted Davidia (hankerchief tree) on the well kept lawn.'

This description may read like the opening paragraph of a pamphlet describing the gardens of some famous historical house now owned by the National Trust. It is, in fact, an accurate (certainly in respect of the trees) description of the western entrance to Southmead Hospital. The gardens at Southmead do not have the instant appeal of those in the more spacious grounds at Ham Green Hospital: but, to the discriminating, if he, or she, is willing to explore; each little bit of the gardens are small gems to be savoured and watched through the seasons.

The gardens have never been better maintained. Despite financial stringencies, the new, young and highly qualified head gardener has accomplished miracles in spite of the constant changes imposed by the erection of new buildings. The lovely purple sycamore growing in front of the new extension to the Pathology Department was not cut down, but transported with tender loving care and now adorns the lawn in front of the Nurses Home. The standard of bedding annuals and shrubs is higher than ever before with scarcely a weed to be seen in the numerous beds throughout the grounds.

The only discordant features are the sight of the greenhouses being allowed to fall down for lack of maintenance and fuel and the fact that nowhere throughout the Southmead Group of Hospitals, which includes several large Mental Hospitals, is there any sight of market gardening, whether of flowers or vegetables. When I raised this point at a Medical Advisory Committee I was told that 'to grow vegetables was not cost effective' – surely a very narrow-minded approach when any 'free vegetables' would cut the catering costs and gardening can be very beneficial as an occupational therapy for mentally disturbed patients.

We are blessed with lovely gardens throughout the hospitals in the Southmead District. It would be nice to think that our patients could benefit not only from the sights and smells in the gardens but also in the healing process of their creation.

